# Novel biomarkers of inflammation in heart failure with preserved ejection fraction: analysis from a large prospective cohort study

**DOI:** 10.1186/s12872-022-02656-z

**Published:** 2022-05-14

**Authors:** Nicholas W. Carris, Rahul Mhaskar, Emily Coughlin, Easton Bracey, Srinivas M. Tipparaju, Ganesh V. Halade

**Affiliations:** 1 Taneja College of Pharmacy, University of South Florida, 12901 Bruce B. Downs Blvd MDC 30, Tampa, FL 33612 USA; 2 Morsani College of Medicine, University of South Florida, 560 Channelside Drive, Tampa, FL 33602 USA

**Keywords:** Heart failure, Inflammation, Interleukin-2, Cardiovascular disease

## Abstract

**Background:**

Heart failure with preserved ejection fraction (HFpEF) is a syndrome with a heterogeneous cluster of causes, including non-resolving inflammation, endothelial dysfunction, and multi-organ defects. The present study’s objective was to identify novel predictors of HFpEF.

**Methods:**

The study analyzed the Multi-Ethnic Study of Atherosclerosis (MESA) to assess the association of specific markers of inflammation with new onset of HFpEF (interleukin-2 [IL-2], matrix metalloproteinase 3 [MMP3], large low-density lipoprotein cholesterol [LDL-C], and medium high-density lipoprotein cholesterol [HDL-C]). The study included men and women 45 to 84 years of age without cardiovascular disease at baseline. The primary outcome was the multivariate association of the hypothesized markers of inflammation with new-onset of HFpEF versus participants without new-onset heart failure. Participants with missing data were excluded.

**Results:**

The present analysis included 6814 participants, 53% female, with a mean age of 62 years. Among the entire cohort, HFpEF was diagnosed in 151 (2.2%) participants and heart failure with reduced ejection fraction (HFrEF) was diagnosed in 146 (2.1%) participants. Participants were followed for the outcome of heart failure for a median 13.9 years. Baseline IL-2 was available for 2861 participants. The multivariate analysis included 2792 participants. Of these, 2668 did not develop heart failure, 62 developed HFpEF, 47 developed HFrEF, and 15 developed unclassified heart failure. In the multivariate regression model, IL-2 was associated with new-onset HFpEF (OR, 1.00058; 95% confidence interval, 1.00014 to 1.00102, *p* = 0.009) but not new-onset HFrEF. In multivariate analysis, MMP3, large LDL-C, and medium HDL-C were not associated with HFpEF or HFrEF.

**Conclusion:**

These findings portend IL-2 as an important component of suboptimal inflammation in the pathogenesis of HFpEF.

**Supplementary Information:**

The online version contains supplementary material available at 10.1186/s12872-022-02656-z.

## Background

Heart failure with reduced ejection fraction (HFrEF) is principally an end-stage of atherosclerosis, while heart failure with preserved ejection fraction (HFpEF) is a syndrome with a heterogeneous cluster of causes, including non-resolving inflammation, endothelial dysfunction, and multi-organ defects [1]. Despite these stark contrasts between HFrEF and HFpEF targeted drug therapy for HFpEF is lacking. Even the recent drug approvals for HFpEF therapy are not directly targeting HFpEF, rather they were first developed for HFrEF or diabetes, and then repositioned for HFpEF [2]. A growing body of literature suggests coronary microvascular disease and endothelial dysfunction may be fundamental contributors to the progression of cardiovascular pathology of HFpEF, with dysregulated inflammation playing a key role in this pathogenesis [3]. However, no current therapies target inflammation or inflammation-related pathway for the prevention of HFpEF.

Acute inflammation plays a key role in host defense in response to myriad other conditions, including infection (covid-19) and injury [4]. However, when acute inflammation remains chronic or dysregulated, it increases the risk for multi-organ inflammatory diseases such as in rheumatoid arthritis, diabetes mellitus, kidney disease, fatty liver disease, and neurodegenerative disorders, and gout [5–7]. Other factors contributing to chronic or dysregulated inflammation include intrinsic patient factors (pro-inflammatory diet, sedentary lifestyle, disrupted sleep wake up cycle) and extrinsic patient factors (smoking/vaping, environmental/noise pollution, and external stress [e.g., psychological stress, depression]) [8, 9]. Aging is a major contributor and risk factor for HFpEF and in combination with frailty, senescence, and other comorbidities magnifies the risk of cardiovascular related deaths [10, 11]. Various cytokines and other makers, such as erythrocyte sedimentation rate and C‐reactive protein, have demonstrated utility in predicting new onset of heart failure. However, they are ill-suited as targeted biomarkers and treatment candidates in HFpEF as they do not differentiate between acute protective inflammation versus suboptimal, and chronic inflammation [12–14].

Rather than simply predicting risk for new onset of HFpEF, the objective of the present study was to identify candidate biomarkers of suboptimal or chronic inflammation potentially suitable for trials of targeted therapy in the prevention of HFpEF. To achieve this objective, we assessed data from the large, prospective cohort study to test biomarker associations with new-onset of HFpEF, new-onset of HFrEF versus no heart failure. We hypothesized that interleukin-2 (IL-2), matrix metalloproteinase 3 (MMP3), large low-density lipoprotein cholesterol (LDL-C), and medium high-density lipoprotein cholesterol (HDL-C) would be independently associated with new-onset HFpEF.

## Methods

The present analysis used data from the Multi-Ethnic Study of Atherosclerosis (MESA) to assess novel biomarkers of dysregulated and chronic inflammation [15]. MESA included participants in the United States aged 45 to 84 years and without clinical cardiovascular disease. MESA was designed to assess patients with subclinical cardiovascular disease at baseline when then did (or did not) go on to develop cardiovascular disease and experience related cardiovascular disease events over the duration of the study. Heart failure was an adjudicated endpoint in MESA. The present analysis labeled participants as having HFpEF with an adjudicated outcome of heart failure with an ejection fraction of at least 45% [16].

The primary outcome of the present study was the multivariate association of four novel biomarkers of inflammatory dysregulation (IL-2, MMP3, large LDL-C, medium HDL-C) with new-onset HFpEF. Associations were tested versus patients without new-onset heart failure to assess the impact on new-onset HFpEF. The biomarkers were also assessed for association with HFrEF and unclassified heart failure to determine if they were specific for HFpEF versus heart failure generally. Univariate and multivariate multinomial logistic regression was used to assess the associations between biomarkers and the outcomes of interest and were summarized as odds ratio (OR) with 95% confidence interval (CI). Models tested the relationship between predictor variables and membership of four groups (no heart failure, HFpEF, HFrEF, and unclassified heart failure, with no heart failure as the reference. For baseline characteristics, the Chi-square test was used to compare categorical variables and Kruskal–Wallis Test for continuous variables. The analysis was hypothesis-driven, and data were not transformed. Participants with missing data were excluded from the regression analysis. We did not impute any missing data. Patients lost to follow-up were considered not to have had an event. To address potential confounding, investigators determined the model a priori based on expected associations between patient characteristics and new-onset HFpEF. No additional subgroups were assessed. Variables included in the model were gender, race, medication use for diabetes, age, urinary albumin creatinine ratio, estimated glomerular filtration rate, systolic blood pressure, pack-years of cigarette smoking, alcohol drinks per week, and body mass index. Of model variables, 1461 participants (21.4%) were missing a value for the number of alcohol drinks per week, the only predictor variable with more than 100 missing data points. The number of alcohol drinks per week was not associated with any outcomes of interest; therefore, the model was re-run without alcohol drinks per week variable. There was no change in significant findings, and the quality of the model was improved. Therefore, a model without the number of alcohol drinks per week is reported herein.

The MESA was started in 2000 with first publication of original MESA study after the informed consent was obtained [15]. The present analysis was performed using MESA Research Materials obtained from the NHLBI Biologic Specimen and Data Repository Information Coordinating Center. The available dataset was last updated 20 November 2017. SPSS version 26 was used for statistical analysis. The study was determined exempt by the University of South Florida Institutional Review Board (STUDY001946). The procedures used in this study adhere to the tenets of the Declaration of Helsinki. Informed consent was obtained in MESA before people were allowed to be MESA participants.

## Results

The present analysis included 6814 participants, 53% female, with a mean age of 62 years (Table 1). Overall, the study population was racially diverse. Relatively few were pharmacologically treated for diabetes at baseline (10% without subsequent HF, 21–24% with subsequent HF), the mean systolic blood pressure was not hypertensive, and on average, participants did not have a significant decrement in renal function. The majority of participants at baseline were never smokers. However, the average pack years of cigarette smoking was higher among patients with HFrEF and unclassified HF compared with patients with HFpEF and no heart failure. HFpEF was diagnosed in 151 (2.2%) participants and HFrEF in 146 (2.1%) participants among the entire cohort. Participants were followed for the outcome of heart failure for a median of 13.93 (3.10) years.

**Table 1:** Baseline characteristics

Characteristic	No HF	HFrEF	HFpEF	Unclassified HF	*p* value
Gender (male)	3012 (46.5)	101 (69.2)	75 (49.7)	25 (59.5)	< 0.001
Age^a^	62.0 (17.0)	68.0 (15.0)	70.0 (12.0)	70.5 (11.0)	< 0.001
BMI^a^	27.5 (6.7)	28.7 (7.1)	28.9 (8.6)	28.5 (8.4)	< 0.001
Race					0.001
Caucasian	2484 (38.4)	58 (39.7)	66 (43.7)	14 (33.3)	0.001
Hispanic	1425 (22.0)	25 (17.1)	36 (23.8)	10 (23.8)	
African American	1786 (27.6)	60 (41.1)	34 (22.5)	12 (28.6)	
Chinese	780 (12.0)	3 (2.1)	15 (9.9)	6 (14.3)	
Education (≤ 12 years)	2318 (35.9)	54 (37.2)	72 (47.7)	17 (40.5)	0.026
Weight^a^	169.5 (51.0)	186.9 (54.3)	180.0 (47.8)	183.4 (72.4)	< 0.001
Seated systolic blood pressure (mmHg)^a^	123.0 (28.5)	133.8 (32.1)	135.5 (31.5)	140.0 (33.1)	< 0.001
Seated diastolic blood pressure (mmHg)^a^	71.5 (13.5)	73.5 (18.0)	72.0 (12.5)	71.8 (14.6)	0.083
Pack-years of cigarette smoking^a^	0.0 (15.0)	5.0 (22.0)	2.4 (21.5)	5.1 (39.3)	0.002
Drinks per week (current and former drinkers)^a^	2.0 (6.0)	2.0 (7.0)	2.5 (7.0)	4.5 (11.0)	0.015
Hypertension medication	2333 (36.0)	83 (56.8)	94 (62.3)	26 (61.9)	< 0.001
Insulin or oral hypoglycemics for diabetes	611 (9.5)	33 (22.9)	35 (23.5)	9 (21.4)	< 0.001
Any lipid-lowering medication	1035 (16.0)	32 (21.9)	26 (17.2)	7 (16.7)	0.279
Total cholesterol (mg/dl)^a^	192.0 (44.0)	190.0 (48.0)	185.0 (48.0)	195.0 (38.0)	0.191
HDL-C (mg/dl)^a^	48.0 (19.0)	44.0 (19.0)	47.0 (16.0)	45.0 (23.0)	0.018
Urinary albumin/creatinine (mg/g)^a^	5.2 (7.2)	8.5 (26.4)	8.4 (37.2)	8.5 (35.3)	< 0.001
Exam 1 (calibrated cr) eGFR using CKD-EPI equation^a^	78.2 (21.7)	73.8 (27.3)	71.9 (24.9)	71.0 (24.6)	< 0.001
Interleukin-2 (pg/ml)^a^	895.0 (422.0)	1033.0 (459.0)	1133.5 (592.0)	1113.0 (617.0)	< 0.001
Matrix metalloproteinase 3 (ng/mL)^a^	11.6 (10.1)	18.3 (14.4)	15.4 (14.5)	19.7 (17.1)	0.011
Large LDL-C 20.5–23 nm (nmol/L) from NMR LipoProfile3 Spectral Analysis^a^	596.0 (342.0)	539.0 (392.0)	547.0 (384.0)	626.5 (451.0)	0.025
Medium HDL-C 8.2–9.4 nm (μmol/L) from NMR LipoProfile3 Spectral Analysis^a^	12.5 (8.5)	10.8 (7.9)	12.0 (9.0)	12.1 (8.4)	0.002
Time to classifying event or follow-up time for patients with No HF (days)^a^	5110 (753)	2335 (2712)	2870 (2253)	1787 (3115)	< 0.001

N (%) or ^a^median (IQR)

*BMI* body mass index, *HF* heart failure, *HDL-C* high-density lipoprotein cholesterol, *HFpEF* heart failure with preserved ejection fraction, *HFrEF* heart failure with a reduced ejection fraction, *LDL-C* low-density lipoprotein cholesterol

Univariate analyses for each variable in the models are reported in the Additional file 1. Baseline IL-2 was available for 2861 participants. In the univariate analysis IL-2 was associated with new-onset HFpEF (OR, 95% CI; 1.00096, 1.00062–1.00129; *p* = 0.01) and new-onset HFrEF (OR, 95% CI; 1.00059, 1.00013–1.00106; *p* < 0.00001). The multivariate analysis included 2792 participants, among which 2668 did not develop heart failure, 62 developed HFpEF, 47 developed HFrEF, and 15 developed heart failure, which could not be classified. In the multivariate regression model, IL-2 remained associated with new-onset of HFpEF (*p* = 0.009) but not new-onset of HFrEF (*p* = 0.34) (Table 2). Overall, the model performed well regarding goodness-of-fit (Pearson, *p* = 1.000; Deviance, *P* = 1.000). In line with this, multiple variables in the model were significantly associated with HFpEF (antidiabetic use, age, pack-years of cigarettes, and body mass index).

**Table 2:** Multivariate model for interleukin-2

HFrEF	Odds ratio	95% Confidence interval		HFpEF	Odds ratio	95% Confidence interval	
Interleukin-2 (pg/ml)	0.99959	0.99873	1.00044	Interleukin-2 (pg/ml)	1.00058	1.00014	1.00102
Gender	3.34842	1.73325	6.46872	Gender	1.63192	0.93732	2.84123
Insulin or oral hypoglycemics for diabetes	3.29388	1.66321	6.52328	Insulin or oral hypoglycemics for diabetes	2.35577	1.26221	4.39678
Age	1.05877	1.02007	1.09893	Age	1.07474	1.03889	1.11183
Urinary albumin/creatine (mg/g)	1.00091	0.99987	1.00194	Urinary albumin/creatine (mg/g)	1.00067	0.99977	1.00157
Exam 1 (calibrated cr) eGFR using CKD-EPI equation	0.99962	0.98012	1.01950	Exam 1 (calibrated cr) eGFR using CKD-EPI equation	1.00374	0.98601	1.02177
Seated systolic blood pressure (mmHg)	1.00870	0.99434	1.02326	Seated systolic blood pressure (mmHg)	1.01044	0.99820	1.02283
Pack years of cigarette smoking	0.98972	0.97252	1.00722	Pack years of cigarette smoking	1.01060	1.00169	1.01960
Body mass index	1.03996	0.97961	1.10402	Body mass index	1.11591	1.06162	1.17296
Hispanic	0.98193	0.40881	2.35851	Hispanic	1.16760	0.56663	2.40595
Chinese	0.24946	0.06510	0.95592	Chinese	1.66794	0.73219	3.79960
African American	1.51587	0.67402	3.40922	African American	0.64135	0.28140	1.46176
White	Ref			White	Ref		

The reference category is no heart failure

*HFpEF* heart failure with preserved ejection fraction, *HFrEF* heart failure with a reduced ejection fraction

Similarly, the model performed well in regard to goodness-of-fit (Pearson, *p* = 1.000; Deviance, *P* = 1.000) when MMP3 (n = 970), large LDL-C (n = 6602), and medium HDL-C (n = 6602) were assessed. In univariate analysis MMP3 was not associated with new-onset HFpEF (OR, 95% CI; 1.016, 0.997–1.035) or new-onset HFrEF (OR, 95% CI; 1.011, 0.990–1.034). However, large LDL-C was associated with HFpEF (OR, 95% CI; 0.99927, 0.99864–0.99989) and HFrEF (OR, 95% CI; 0.99923, 0.99860–0.99987). Medium HDL-C was only associated with HFrEF; HFpEF (OR, 95% CI; 0.988, 0.964–1.013) and HFrEF (OR, 95% CI; 0.95207, 0.92646–0.97838). However, MMP3, large LDL-C, and medium HDL-C were not associated with HFpEF or HFrEF in multivariate analysis (Tables 3, 4, 5).

**Table 3:** Multivariate model for matrix metalloproteinase 3

HFrEF	Odds ratio	95% Confidence interval		HFpEF	Odds ratio	95% Confidence interval	
Matrix metalloproteinase 3	1.00127	0.96734	1.03639	Matrix metalloproteinase 3	1.01385	0.98936	1.03894
Gender	2.90090	0.96374	8.73185	Gender	0.97167	0.31312	3.01526
Insulin or oral hypoglycemics for diabetes	3.34869	0.79411	14.12100	Insulin or oral hypoglycemics for diabetes	8.31555	2.51490	27.49543
Age	1.04987	0.98472	1.11934	Age	1.14281	1.06540	1.22584
Urinary albumin/creatine (mg/g)	0.99538	0.97905	1.01198	Urinary albumin/creatine (mg/g)	1.00151	0.99987	1.00316
Exam 1 (calibrated cr) eGFR using CKD-EPI equation	1.01781	0.98069	1.05633	Exam 1 (calibrated cr) eGFR using CKD-EPI equation	1.03743	1.00389	1.07209
Seated systolic blood pressure (mmHg)	1.03456	1.01094	1.05873	Seated systolic blood pressure (mmHg)	0.99732	0.97237	1.02290
Pack years of cigarette smoking	0.99277	0.96718	1.01903	Pack years of cigarette smoking	1.01813	1.00610	1.03030
Body mass index	0.99635	0.90042	1.10249	Body mass index	1.15811	1.05560	1.27057
Hispanic	0.42105	0.08233	2.15337	Hispanic	0.29495	0.06149	1.41490
Chinese	< 0.00001	< 0.00001	< 0.00001	Chinese	0.65042	0.04964	8.52208
African American	1.39120	0.42897	4.51178	African American	0.25390	0.05720	1.12701
White	Ref			White	Ref		

The reference category is no heart failure

*HFpEF* heart failure with preserved ejection fraction, *HFrEF* heart failure with a reduced ejection fraction

**Table 4:** Multivariate model for large low-density lipoprotein cholesterol

HFrEF	Odds ratio	95% Confidence interval		HFpEF	Odds ratio	95% Confidence interval	
Large low-density lipoprotein cholesterol	0.21995	0.99956	0.99885	Large low-density lipoprotein cholesterol	0.16545	0.99952	0.99883
Gender	0.00000	2.69765	1.82503	Gender	0.38824	1.17238	0.81691
Insulin or oral hypoglycemics for diabetes	0.00161	2.04545	1.31108	Insulin or oral hypoglycemics for diabetes	0.00671	1.83059	1.18229
Age	0.00010	1.04290	1.02108	Age	0.00000	1.07398	1.05073
Urinary albumin/creatine (mg/g)	0.00665	1.00065	1.00018	Urinary albumin/creatine (mg/g)	0.00583	1.00068	1.00020
Exam 1 (calibrated cr) eGFR using CKD-EPI equation	0.44970	0.99566	0.98451	Exam 1 (calibrated cr) eGFR using CKD-EPI equation	0.32205	1.00582	0.99433
Seated systolic blood pressure (mmHg)	0.00230	1.01251	1.00445	Seated systolic blood pressure (mmHg)	0.00026	1.01428	1.00660
Pack years of cigarette smoking	0.32186	0.99589	0.98781	Pack years of cigarette smoking	0.06889	1.00504	0.99961
Body mass index	0.21234	1.02285	0.98717	Body mass index	0.00001	1.07556	1.04117
Hispanic	0.03581	0.57121	0.33861	Hispanic	0.18962	0.73984	0.47163
Chinese	0.00260	0.16294	0.05003	Chinese	0.76581	0.91225	0.49850
African American	0.25676	1.25814	0.84600	African American	0.00298	0.49707	0.31336
White	Ref			White	Ref		

The reference category is no heart failure

*HFpEF* heart failure with preserved ejection fraction, *HFrEF* heart failure with a reduced ejection fraction

**Table 5:** Multivariate model for medium high-density lipoprotein cholesterol

HFrEF	Odds ratio	95% Confidence interval		HFpEF	Odds ratio	95% Confidence interval	
Medium high-density lipoprotein cholesterol	0.18855	0.98020	0.95141	Medium high-density lipoprotein cholesterol	0.85785	1.00245	0.97592
Gender	0.00000	2.68928	1.82156	Gender	0.20829	1.26513	0.87709
Insulin or oral hypoglycemics for diabetes	0.00116	2.07720	1.33621	Insulin or oral hypoglycemics for diabetes	0.00345	1.91425	1.23877
Age	0.00015	1.04176	1.01994	Age	0.00000	1.07372	1.05044
Urinary albumin/creatine (mg/g)	0.00966	1.00063	1.00015	Urinary albumin/creatine (mg/g)	0.00637	1.00067	1.00019
Exam 1 (calibrated cr) eGFR using CKD-EPI equation	0.49541	0.99606	0.98482	Exam 1 (calibrated cr) eGFR using CKD-EPI equation	0.34952	1.00555	0.99396
Seated systolic blood pressure (mmHg)	0.00194	1.01274	1.00467	Seated systolic blood pressure (mmHg)	0.00025	1.01431	1.00663
Pack years of cigarette smoking	0.33463	0.99600	0.98792	Pack years of cigarette smoking	0.06988	1.00509	0.99959
Body mass index	0.19655	1.02351	0.98804	Body mass index	0.00000	1.07933	1.04515
Hispanic	0.03331	0.56655	0.33573	Hispanic	0.20768	0.74832	0.47667
Chinese	0.00214	0.15632	0.04780	Chinese	0.90282	0.96245	0.52072
African American	0.39394	1.19014	0.79761	African American	0.00261	0.48868	0.30659
White	Ref			White	Ref		

The reference category is no heart failure

*HFpEF* heart failure with preserved ejection fraction, *HFrEF* heart failure with a reduced ejection fraction

## Discussion

The present analysis identified IL-2 as a biomarker associated with, and a potential therapeutic target for, preventing the progression to HFpEF. The study did not identify LDL-C or HDL-C particle size as associated with HFpEF. While the present study did not evaluate the direct roles of oxLDL-C, previous reports have assessed the relation of LDL-C and HFpEF [17]. Moreover, prior reports have investigated the relation between heart failure and HDL-C and LDL-C [18]. Therefore, while correlation likely exists between HDL-C/LDL-C overall, the present analysis was primarily assessing linkage between inflammation and HFpEF. In regard to MMP3, it was not associated with HFpEF herein, however there was a larger proportion of missing data than other assessed biomarkers. Additionally, the OR trended toward a higher odds of HFpEF. As such, future studies with adequate data to assess MMP3 may find an association with HFpEF where the current study did not.

Diagnosis of HFpEF is frequently challenging and heterogeneous in nature, however the present analysis used data from a high-quality prospective cohort study [15]. The present study assessed ejection fraction based on a cut point of 45% for HFpEF versus HFrEF. The recently released 2022 heart failure guidelines created a new formal category of heart failure, “heart failure with mildly reduced ejection fraction” including ejection fraction percentages from 41 to 49% [19]. The present study elected not to reclassify as the new classification has not be specifically implemented in randomized controlled trials and reclassifying to a third class of heart failure would have only generated 33 unique incident cases. Thus, the present study relied on the traditional cut point of 45%. In regard to data analysis, a strength of the present study is that it was hypothesis-driven and completed without data imputation or data transformation [20]. The approach taken is the most conservative regarding novel outcome predictors in cohort studies. Additionally, the association of IL-2 with new-onset HFpEF was identified despite relatively few cases of new-onset HFpEF. One weakness of the analysis is the OR identified for IL-2 was relatively small. However, this is explained by the overall low event rate. Additionally, the lack of association with HFrEF lends credence to the association found with HFpEF. Indeed, HFrEF and HFpEF have differing pathophysiology, specifically, HFpEF versus HFrEF is more likely to have endothelial dysfunction and concentric rather than eccentric hypertrophy, and less likely to have cardiomyocyte cell death [21]. Along these pathophysiologic lines, inflammation appears to play a role in the pathogenesis of HFpEF but not (or much less so) HFrEF. Other weaknesses of the present study include missing data, though handled most conservatively [3]. Additionally, IL-2 was only measured at baseline; therefore, we were unable to assess total exposure to IL-2 or chronic inflammation. Finally, the study was observational; therefore, causal inference cannot be made.

A recent study assessed IL-2 levels and correlated with new onset heart failure using MESA data [22]. However, this study did not distinguish between HFpEF and HFrEF. This study also found an association of IL-2 with heart failure. In the present, hypothesis driven study, without data transformation, we directly pointed the association of IL-2 at HFpEF, without association with HFrEF. Thus, helping to differentiate the heterogeneous pathophysiology, broadly split between HFpEF and HFrEF. The present study, however, was not able to classify every incident case of heart failure, as 42 patients in the entire cohort had probable or definite heart failure that could not be classified as HFpEF or HFrEF. Importantly, our results do not conflict with a recent study of IL-2 in myocardial healing [23]. Indeed, appropriate acute inflammation response is vital in multiple aspects of host defense and cardiac repair in contrast to sustained higher levels of inflammatory mediators and inflammation in aging [24, 25]. As the current analysis addressed baseline IL-2 in patients without cardiovascular disease, our results indicate IL-2 is a suboptimal and chronic inflammation component.

## Conclusions

The present analysis is the first study identifying IL-2 as predictive of new-onset HFpEF. These findings portend IL-2 as an important component of suboptimal inflammation in the pathogenesis of HFpEF. Additional mechanistic and clinical studies are needed to fully elucidate this finding and a potential relationship between the role of IL-2 in acute inflammation (helpful) and suboptimal inflammation of coronary microvascular disease (harmful). Future research needs to address IL-2, chronic inflammation, and failure of acute inflammation to resolve concerning the prevention and treatment of HFpEF.

## Supplementary Information

**Additional file 1.** Appendix 1: Univariate analyses.

## Data Availability

The datasets generated and/or analysed during the current study are available in the National Heart, Lung, and Blood Institute’s Biologic Specimen and Data Repository Information Coordinating Center repository, https://biolincc.nhlbi.nih.gov/studies/mesa/.

## Abbreviations

CI: Confidence interval
HDL-C: High-density lipoprotein cholesterol
HFpEF: Heart failure with preserved ejection fraction
HFrEF: Heart failure with reduced ejection fraction
IL-2: Interleukin-2
LDL-C: Low-density lipoprotein cholesterol
MESA: Multi-Ethnic Study of Atherosclerosis
MMP3: Matrix metalloproteinase 3
OR: Odds ratio
