# Supplementary material for: Novel biomarkers of inflammation in heart failure with preserved ejection fraction: analysis from a large prospective cohort study
Source: BMC Cardiovasc Disord. 2022 May 14;22:221. doi: 10.1186/s12872-022-02656-z (PMC9107006; doi:10.1186/s12872-022-02656-z)
Supplement: Supplementary file 1 — Additional file 1. Appendix 1: Univariate analyses. [file 12872_2022_2656_MOESM1_ESM.docx]

**Appendix 1: Univariate analyses**

**Table S1: Univariate Analysis: Race/Ethnicity**

| **Parameter Estimates** | | | | | | | | | |
| --- | --- | --- | --- | --- | --- | --- | --- | --- | --- |
| HFpEF or HFrEF^a^ | | B | Std. Error | Wald | df | Sig. | Exp(B) | 95% Confidence Interval for Exp(B) | |
|  |  |  |  |  |  |  |  | Lower Bound | Upper Bound |
| HFrEF | Intercept | -3.757 | 0.133 | 800.071 | 1 | 0.000 |  |  |  |
|  | [Race = Hispanic] | -0.286 | 0.242 | 1.401 | 1 | 0.237 | 0.751 | 0.468 | 1.206 |
|  | [Race Chinese] | -1.803 | 0.594 | 9.234 | 1 | 0.002 | 0.165 | 0.051 | 0.527 |
|  | [Race = African American] | 0.364 | 0.187 | 3.795 | 1 | 0.051 | 1.439 | 0.998 | 2.075 |
|  | [Race = White] | 0^b^ |  |  | 0 |  |  |  |  |
| HFpEF | Intercept | -3.628 | 0.125 | 846.219 | 1 | 0.000 |  |  |  |
|  | [Race = Hispanic] | -0.050 | 0.210 | 0.058 | 1 | 0.810 | 0.951 | 0.630 | 1.435 |
|  | [Race Chinese] | -0.323 | 0.289 | 1.252 | 1 | 0.263 | 0.724 | 0.411 | 1.275 |
|  | [Race = African American] | -0.333 | 0.213 | 2.442 | 1 | 0.118 | 0.716 | 0.472 | 1.088 |
|  | [Race = White] | 0^b^ |  |  | 0 |  |  |  |  |
| HF Unclassified | Intercept | -5.179 | 0.268 | 373.342 | 1 | 0.000 |  |  |  |
|  | [Race = Hispanic] | 0.219 | 0.415 | 0.279 | 1 | 0.598 | 1.245 | 0.552 | 2.810 |
|  | [Race Chinese] | 0.311 | 0.490 | 0.403 | 1 | 0.525 | 1.365 | 0.523 | 3.564 |
|  | [Race = African American] | 0.176 | 0.395 | 0.198 | 1 | 0.656 | 1.192 | 0.550 | 2.584 |
|  | [Race = White] | 0^b^ |  |  | 0 |  |  |  |  |
|  | | | | | | | | | |
|  | | | | | | | | | |

**Table S2: Univariate Analysis: Gender**

| **Parameter Estimates** | | | | | | | | | |
| --- | --- | --- | --- | --- | --- | --- | --- | --- | --- |
| HFpEF or HFrEF^a^ | | B | Std. Error | Wald | df | Sig. | Exp(B) | 95% Confidence Interval for Exp(B) | |
|  |  |  |  |  |  |  |  | Lower Bound | Upper Bound |
| HFrEF | Intercept | -4.343 | 0.150 | 837.974 | 1 | 0.000 |  |  |  |
|  | Gender | 0.948 | 0.181 | 27.446 | 1 | 0.000 | 2.581 | 1.810 | 3.679 |
| HFpEF | Intercept | -3.819 | 0.116 | 1084.727 | 1 | 0.000 |  |  |  |
|  | Gender | 0.126 | 0.165 | 0.588 | 1 | 0.443 | 1.135 | 0.822 | 1.567 |
| HF Unclassified | Intercept | -5.317 | 0.243 | 478.192 | 1 | 0.000 |  |  |  |
|  | Gender | 0.525 | 0.315 | 2.774 | 1 | 0.096 | 1.691 | 0.911 | 3.137 |

**Table S3: Univariate Analysis: Age**

| **Parameter Estimates** | | | | | | | | | | |
| --- | --- | --- | --- | --- | --- | --- | --- | --- | --- | --- |
| HFpEF or HFrEF^a^ | | | B | Std. Error | Wald | df | Sig. | Exp(B) | 95% Confidence Interval for Exp(B) | |
|  |  |  |  |  |  |  |  |  | Lower Bound | Upper Bound |
| HFrEF | Intercept | -7.251 | | 0.582 | 155.412 | 1 | 0.000 |  |  |  |
|  | Age | 0.054 | | 0.009 | 39.042 | 1 | 0.000 | 1.055 | 1.037 | 1.073 |
| HFpEF | Intercept | -8.445 | | 0.611 | 190.820 | 1 | 0.000 |  |  |  |
|  | Age | 0.072 | | 0.009 | 66.030 | 1 | 0.000 | 1.074 | 1.056 | 1.093 |
| HF Unclassified | Intercept | -10.795 | | 1.222 | 78.000 | 1 | 0.000 |  |  |  |
|  | Age | 0.087 | | 0.017 | 25.335 | 1 | 0.000 | 1.091 | 1.055 | 1.128 |

**Table S4: Univariate Analysis: Insulin or Oral Hypoglycemics for Diabetes**

| **Parameter Estimates** | | | | | | | | | |
| --- | --- | --- | --- | --- | --- | --- | --- | --- | --- |
| HFpEF or HFrEF^a^ | | B | Std. Error | Wald | df | Sig. | Exp(B) | 95% Confidence Interval for Exp(B) | |
|  |  |  |  |  |  |  |  | Lower Bound | Upper Bound |
| HFrEF | Intercept | -3.958 | 0.096 | 1706.749 | 1 | 0.000 |  |  |  |
|  | Insulin or Oral Hypoglycemics for Diabetes | 1.040 | 0.203 | 26.298 | 1 | 0.000 | 2.829 | 1.901 | 4.210 |
| HFpEF | Intercept | -3.932 | 0.095 | 1728.463 | 1 | 0.000 |  |  |  |
|  | Insulin or Oral Hypoglycemics for Diabetes | 1.072 | 0.198 | 29.356 | 1 | 0.000 | 2.921 | 1.982 | 4.305 |
| HF Unclassified | Intercept | -5.172 | 0.175 | 877.590 | 1 | 0.000 |  |  |  |
|  | Insulin or Oral Hypoglycemics for Diabetes | 0.954 | 0.378 | 6.350 | 1 | 0.012 | 2.595 | 1.236 | 5.449 |

**Table S5: Univariate Analysis: Urinary Albumin Creatine Ratio**

| **Parameter Estimates** | | | | | | | | | |
| --- | --- | --- | --- | --- | --- | --- | --- | --- | --- |
| HFpEF or HFrEF^a^ | | B | Std. Error | Wald | df | Sig. | Exp(B) | 95% Confidence Interval for Exp(B) | |
|  |  |  |  |  |  |  |  | Lower Bound | Upper Bound |
| HFrEF | Intercept | -3.843 | 0.086 | 2005.416 | 1 | 0.000 |  |  |  |
|  | Urinary Albumin/Creatine (mg/g) | 0.001 | 0.000 | 20.077 | 1 | 0.000 | 1.001 | 1.001 | 1.001 |
| HFpEF | Intercept | -3.816 | 0.085 | 2033.252 | 1 | 0.000 |  |  |  |
|  | Urinary Albumin/Creatine (mg/g) | 0.001 | 0.000 | 24.807 | 1 | 0.000 | 1.001 | 1.001 | 1.002 |
| HF Unclassified | Intercept | -5.077 | 0.157 | 1039.071 | 1 | 0.000 |  |  |  |
|  | Urinary Albumin/Creatine (mg/g) | 0.001 | 0.000 | 10.010 | 1 | 0.002 | 1.001 | 1.000 | 1.002 |

**Table S6: Univariate Analysis: Exam 1 (Calibrated cr) eGFR Using CKD-EPI Equation**

| **Parameter Estimates** | | | | | | | | | |
| --- | --- | --- | --- | --- | --- | --- | --- | --- | --- |
| HFpEF or HFrEF^a^ | | B | Std. Error | Wald | df | Sig. | Exp(B) | 95% Confidence Interval for Exp(B) | |
|  |  |  |  |  |  |  |  | Lower Bound | Upper Bound |
| HFrEF | Intercept | -1.992 | 0.369 | 29.224 | 1 | 0.000 |  |  |  |
|  | Exam 1 (Calibrated cr) eGFR Using CKD-EPI Equation | -0.024 | 0.005 | 23.011 | 1 | 0.000 | 0.976 | 0.967 | 0.986 |
| HFpEF | Intercept | -2.104 | 0.366 | 32.974 | 1 | 0.000 |  |  |  |
|  | Exam 1 (Calibrated cr) eGFR Using CKD-EPI Equation | -0.022 | 0.005 | 19.787 | 1 | 0.000 | 0.978 | 0.969 | 0.988 |
| HF Unclassified | Intercept | -2.966 | 0.663 | 20.043 | 1 | 0.000 |  |  |  |
|  | Exam 1 (Calibrated cr) eGFR Using CKD-EPI Equation | -0.028 | 0.009 | 9.283 | 1 | 0.002 | 0.973 | 0.955 | 0.990 |

**Table S7: Univariate Analysis: Seated Systolic Blood Pressure (mm HG)**

| **Parameter Estimates** | | | | | | | | | |
| --- | --- | --- | --- | --- | --- | --- | --- | --- | --- |
| HFpEF or HFrEF^a^ | | B | Std. Error | Wald | df | Sig. | Exp(B) | 95% Confidence Interval for Exp(B) | |
|  |  |  |  |  |  |  |  | Lower Bound | Upper Bound |
| HFrEF | Intercept | -6.330 | 0.480 | 174.225 | 1 | 0.000 |  |  |  |
|  | Seated Systolic Blood Pressure (mmHg) | 0.019 | 0.003 | 31.097 | 1 | 0.000 | 1.020 | 1.013 | 1.027 |
| HFpEF | Intercept | -7.173 | 0.468 | 235.001 | 1 | 0.000 |  |  |  |
|  | Seated Systolic Blood Pressure (mmHg) | 0.026 | 0.003 | 60.729 | 1 | 0.000 | 1.026 | 1.019 | 1.033 |
| HF Unclassified | Intercept | -8.256 | 0.873 | 89.373 | 1 | 0.000 |  |  |  |
|  | Seated Systolic Blood Pressure (mmHg) | 0.024 | 0.006 | 15.487 | 1 | 0.000 | 1.025 | 1.012 | 1.037 |

**Table S8: Univariate Analysis: Pack Years of Cigarette Smoking**

| **Parameter Estimates** | | | | | | | | | |
| --- | --- | --- | --- | --- | --- | --- | --- | --- | --- |
| HFpEF or HFrEF^a^ | | B | Std. Error | Wald | df | Sig. | Exp(B) | 95% Confidence Interval for Exp(B) | |
|  |  |  |  |  |  |  |  | Lower Bound | Upper Bound |
| HFrEF | Intercept | -3.852 | 0.095 | 1637.741 | 1 | 0.000 |  |  |  |
|  | Pack Years of Cigarette Smoking | 0.004 | 0.003 | 1.728 | 1 | 0.189 | 1.004 | 0.998 | 1.011 |
| HFpEF | Intercept | -3.853 | 0.092 | 1754.113 | 1 | 0.000 |  |  |  |
|  | Pack Years of Cigarette Smoking | 0.007 | 0.003 | 7.849 | 1 | 0.005 | 1.007 | 1.002 | 1.012 |
| HF Unclassified | Intercept | -5.185 | 0.167 | 962.007 | 1 | 0.000 |  |  |  |
|  | Pack Years of Cigarette Smoking | 0.010 | 0.003 | 12.354 | 1 | 0.000 | 1.011 | 1.005 | 1.016 |

**Table S9: Univariate Analysis: BMI**

| **Parameter Estimates** | | | | | | | | | |
| --- | --- | --- | --- | --- | --- | --- | --- | --- | --- |
| HFpEF or HFrEF^a^ | | B | Std. Error | Wald | df | Sig. | Exp(B) | 95% Confidence Interval for Exp(B) | |
|  |  |  |  |  |  |  |  | Lower Bound | Upper Bound |
| HFrEF | Intercept | -4.735 | 0.429 | 121.587 | 1 | 0.000 |  |  |  |
|  | BMI | 0.033 | 0.014 | 5.186 | 1 | 0.023 | 1.033 | 1.005 | 1.063 |
| HFpEF | Intercept | -5.414 | 0.411 | 173.312 | 1 | 0.000 |  |  |  |
|  | BMI | 0.057 | 0.013 | 17.985 | 1 | 0.000 | 1.058 | 1.031 | 1.087 |
| HF Unclassified | Intercept | -7.092 | 0.760 | 87.128 | 1 | 0.000 |  |  |  |
|  | BMI | 0.070 | 0.024 | 8.286 | 1 | 0.004 | 1.072 | 1.023 | 1.125 |

**Table S10: Univariate Analysis: Interleukin-2 (pg/ml)**

| **Parameter Estimates** | | | | | | | | | |
| --- | --- | --- | --- | --- | --- | --- | --- | --- | --- |
| HFpEF or HFrEF^a^ | | B | Std. Error | Wald | df | Sig. | Exp(B) | 95% Confidence Interval for Exp(B) | |
|  |  |  |  |  |  |  |  | Lower Bound | Upper Bound |
| HFrEF | Intercept | -4.582 | 0.301 | 230.992 | 1 | 0.000 |  |  |  |
|  | Interleukin-2 (pg/ml) | 0.001 | 0.000 | 6.205 | 1 | 0.013 | 1.001 | 1.000 | 1.001 |
| HFpEF | Intercept | -4.813 | 0.248 | 376.015 | 1 | 0.000 |  |  |  |
|  | Interleukin-2 (pg/ml) | 0.001 | 0.000 | 31.876 | 1 | 0.000 | 1.001 | 1.001 | 1.001 |
| HF Unclassified | Intercept | -5.847 | 0.548 | 113.884 | 1 | 0.000 |  |  |  |
|  | Interleukin-2 (pg/ml) | 0.001 | 0.000 | 2.064 | 1 | 0.151 | 1.001 | 1.000 | 1.001 |

**Table S11: Univariate Analysis: Matrix Metalloproteinase 3**

| **Parameter Estimates** | | | | | | | | | | | |
| --- | --- | --- | --- | --- | --- | --- | --- | --- | --- | --- | --- |
| HFpEF or HFrEF^a^ | | | B | | Std. Error | Wald | df | Sig. | Exp(B) | 95% Confidence Interval for Exp(B) | |
|  |  |  |  |  |  |  |  |  |  | Lower Bound | Upper Bound |
| HFrEF | Intercept | -4.110 | | 0.312 | | 173.559 | 1 | 0.000 |  |  |  |
|  | Matrix Metalloproteinase 3 (ng/mL) | 0.011 | | 0.011 | | 1.030 | 1 | 0.310 | 1.011 | 0.990 | 1.034 |
| HFpEF | Intercept | -4.192 | | 0.306 | | 187.875 | 1 | 0.000 |  |  |  |
|  | Matrix Metalloproteinase 3 (ng/mL) | 0.015 | | 0.010 | | 2.564 | 1 | 0.109 | 1.016 | 0.997 | 1.035 |
| HF Unclassified | Intercept | -5.411 | | 0.610 | | 78.724 | 1 | 0.000 |  |  |  |
|  | Matrix Metalloproteinase 3 (ng/mL) | 0.009 | | 0.023 | | 0.172 | 1 | 0.678 | 1.010 | 0.965 | 1.056 |

**Table S12: Univariate Analysis: Large LDL 20.5-23 nm (nmol/L) from NMR LipoProfile3 Spectral Analysis**

| **Parameter Estimates** | | | | | | | | | |
| --- | --- | --- | --- | --- | --- | --- | --- | --- | --- |
| HFpEF or HFrEF^a^ | | B | Std. Error | Wald | df | Sig. | Exp(B) | 95% Confidence Interval for Exp(B) | |
|  |  |  |  |  |  |  |  | Lower Bound | Upper Bound |
| HFrEF | Intercept | -3.356 | 0.193 | 301.047 | 1 | 0.000 |  |  |  |
|  | Large LDL 20.5-23 nm (nmol/L) from NMR LipoProfile3 Spectral Analysis | -0.001 | 0.000 | 5.612 | 1 | 0.018 | 0.999 | 0.999 | 1.000 |
| HFpEF | Intercept | -3.346 | 0.191 | 305.413 | 1 | 0.000 |  |  |  |
|  | Large LDL 20.5-23 nm (nmol/L) from NMR LipoProfile3 Spectral Analysis | -0.001 | 0.000 | 5.289 | 1 | 0.021 | 0.999 | 0.999 | 1.000 |
| HF Unclassified | Intercept | -4.873 | 0.375 | 169.200 | 1 | 0.000 |  |  |  |
|  | Large LDL 20.5-23 nm (nmol/L) from NMR LipoProfile3 Spectral Analysis | 0.000 | 0.001 | 0.215 | 1 | 0.643 | 1.000 | 0.999 | 1.001 |

**Table S13: Univariate Analysis: Medium HDL 8.2-9.4 nm (μmol/L) from NMR LipoProfile3 Spectral Analysis**

| **Parameter Estimates** | | | | | | | | | |
| --- | --- | --- | --- | --- | --- | --- | --- | --- | --- |
| HFpEF or HFrEF^a^ | | B | Std. Error | Wald | df | Sig. | Exp(B) | 95% Confidence Interval for Exp(B) | |
|  |  |  |  |  |  |  |  | Lower Bound | Upper Bound |
| HFrEF | Intercept | -3.185 | 0.179 | 317.514 | 1 | 0.000 |  |  |  |
|  | Medium HDL 8.2-9.4 nm (μmol/L) from NMR LipoProfile3 Spectral Analysis | -0.049 | 0.014 | 12.467 | 1 | 0.000 | 0.952 | 0.926 | 0.978 |
| HFpEF | Intercept | -3.606 | 0.180 | 402.422 | 1 | 0.000 |  |  |  |
|  | Medium HDL 8.2-9.4 nm (μmol/L) from NMR LipoProfile3 Spectral Analysis | -0.012 | 0.012 | 0.909 | 1 | 0.340 | 0.988 | 0.964 | 1.013 |
| HF Unclassified | Intercept | -4.820 | 0.336 | 205.724 | 1 | 0.000 |  |  |  |
|  | Medium HDL 8.2-9.4 nm (μmol/L) from NMR LipoProfile3 Spectral Analysis | -0.016 | 0.024 | 0.484 | 1 | 0.487 | 0.984 | 0.939 | 1.030 |
